# The role of Mannose Binding Lectin in the immune response against *Borrelia burgdorferi* sensu lato

**DOI:** 10.1038/s41598-018-37922-8

**Published:** 2019-02-05

**Authors:** Jeroen Coumou, Alex Wagemakers, Sukanya Narasimhan, Tim J. Schuijt, Jasmin I. Ersoz, Anneke Oei, Onno J. de Boer, Joris J. T. H. Roelofs, Erol Fikrig, Joppe W. Hovius

**Affiliations:** 1 Amsterdam UMC, University of Amsterdam, Center for Experimental and Molecular Medicine, Meibergdreef 9, Amsterdam, Netherlands; 2 Department of Internal Medicine, Yale University School of Medicine, 06511 New Haven, CT USA; 3 Amsterdam UMC, University of Amsterdam, Department of Medical Microbiology, Meibergdreef 9, Amsterdam, Netherlands; 4 Amsterdam UMC, University of Amsterdam, Department of Pathology, Amsterdam Cardiovascular Sciences, Amsterdam Infection & Immunity, Meibergdreef 9, Amsterdam, Netherlands

## Abstract

The causative agents of Lyme borreliosis, spirochetes belonging to the *Borrelia burgdorferi* sensu lato group, have developed several ways to protect themselves against killing by the host complement system. In addition, it has been shown that serum sensitive isolates are (partially) protected by the *Ixodes* Tick Salivary Lectin Pathway Inhibitor (TSLPI) protein; a salivary gland protein that inhibits the function of Mannose Binding Lectin (MBL). MBL is a C-type lectin that recognizes oligosaccharides on pathogens and activates the complement system via the lectin pathway. MBL deficiency has been linked to a more severe course of several infectious diseases and humans with detectable antibodies against *B. burgdorferi* are significantly more often MBL deficient compared to humans without antibodies against *B. burgdorferi*. Here we set out to investigate the role of MBL in the immune response against *B. burgdorferi* in more detail. We demonstrate that *B. burgdorferi* N40 needle-infected C57BL/6 MBL deficient mice harbored significantly higher *B. burgdorferi* numbers in skin tissue during the early course of infection. In line with these findings they also developed higher anti-*B. burgdorferi* IgG serum antibodies compared to WT controls. In contrast*, B. burgdorferi* loads in distant tissue such as heart, joints or bladder at later time points were similar for both mouse strains. These *in vivo* findings were corroborated using a *B. burgdorferi* N40-infected *I. scapularis* infestation model. We showed that MBL is capable of binding *B. burgdorferi* through its carbohydrate recognition domains, but *in vitro* complement killing assays, peritoneal macrophage and whole blood stimulations, phagocytosis assays and an *in vivo* migration experiment did not reveal the mechanism by which MBL facilitates early clearance of *B. burgdorferi*. To conclude, we show a protective role of MBL in the early stages of *B. burgdorferi* infection, yet the underlying mechanism warrants further investigation.

## Introduction

The causative agents of Lyme borreliosis (LB), spirochetes belonging to the *Borrelia burgdorferi* sensu lato group, are transmitted by *Ixodes* ticks^1^. Since 1981 multiple *Borrelia* species have been identified as the causative agents of LB and are being referred to as *B. burgdorferi* sensu lato (s.l.). In the USA, *B. burgdorferi* is the predominant prevalent agent for LB, whereas in Europe *B. afzelii* and *B. garinii* are the main causative agents of LB^2,3^. Infection with *B. burgdorferi* s.l. can lead to erythema migrans (EM) at the tick bite site after which dissemination to other skin sites or to the heart, joint or the central nervous system can occur^4^.

During transmission and dissemination, *B. burgdorferi* s.l. has developed several ways to evade and modulate the host’s innate and adaptive immune responses^5^. Among these mechanisms are the exploitation of immunosuppressive tick proteins and the ability to suppress activation of the complement system^6^. The complement system consists of approximately 30 proteins and its main functions are killing of pathogens by lysis through the membrane attack complex (MAC), activation and attraction of leukocytes and opsonisation of pathogens for phagocytosis by leukocytes^7^. The complement system can be activated via three different pathways; the classical pathway, the lectin pathway and the alternative pathway. *B. burgdorferi* s.l. is able to suppress activation of the complement system via expression of complement regulation proteins on its extracellular membrane such as complement regulator acquiring surface proteins (CRASPs), CD59 like proteins and C4b-binding proteins^6,8–10^. The expression of these proteins varies between different *B. burgdorferi* s.l. strains, making some strains more susceptible to eradication by the complement system than others^11^.

We have previously demonstrated the role of the lectin pathway in the immune response against *B. burgdorferi* by the identification and characterisation of the tick salivary gland protein Tick Salivary Lectin Pathway Inhibitor (TSLPI)^12^. *In vitro* experiments revealed that *I. scapularis* as well as *I. ricinus* TSLPI was able to specifically inhibit the lectin pathway by binding to MBL - resulting in decreased complement-dependent killing of *B. burgdorferi* by human serum^12,13^. Furthermore, phagocytosis of *B. burgdorferi* by human neutrophils was decreased, as well as *B. burgdorferi* transmission to mice by ticks in which TSLPI was silenced.

MBL is a soluble pattern-recognition molecule, which can activate the lectin pathway after binding to oligosaccharides on the surface of pathogens. Furthermore, opsonisation of MBL to pathogens initiates phagocytosis by leukocytes, as well as cytokine responses independent of complement activation^14^. Around 20–25% of the human population is MBL deficient or has low MBL levels (defined as <500 ng/mL in serum)^15,16^. Studies have shown that MBL deficiency can result in increased susceptibility to infections, in particular in patients in whom the adaptive immune system is suppressed or immature^17^. On the contrary, MBL deficiency also seems to have a protective role against adversarial effects of the immune response, such as tissue damage caused by activation of the complement system^18^. Others have shown that MBL deficiency correlated with the presence of antibodies against *B. burgdorferi* s.l. in human sera^19^. Although seropositivity for antibodies against *B. burgdorferi* s.l. does not necessarily indicate active Lyme borreliosis, these findings support the theory that MBL deficient individuals have a higher risk of contracting LB.

To elucidate the role of MBL deficiency in the immune response against *B. burgdorferi* s.l. we performed experiments with wildtype (WT) C57BL/6 mice and a C57BL/6 mouse strain that is deficient for MBL^20^. Whereas humans express one type of MBL, mice express two types of MBL, namely MBL-A and MBL-C^21^. Studies with the double knock-out MBL-A and -C deficient mice have shown the protective role of MBL against several pathogens, *i.e. S. aureus, P. aeruginosa, C. albicans* and others, as well as the benefit of MBL deficiency in tissue injury caused by complement activation^22^. In this study we infected wild type (WT) C57BL/6 mice and double knock-out MBL-A and -C C57BL/6 mice with *B. burgdorferi* and monitored *B. burgdorferi* tissue loads and histopathological changes to determine the role of MBL *in vivo*. Furthermore, we set out to determine the role of MBL deficiency in specific innate immune responses against *B. burgdorferi*, such as complement killing assays, peritoneal macrophage and whole blood stimulations, phagocytosis assays and *in vivo* migration assays.

## Results

### MBL levels in LB patients versus healthy controls

**Figure 1:**
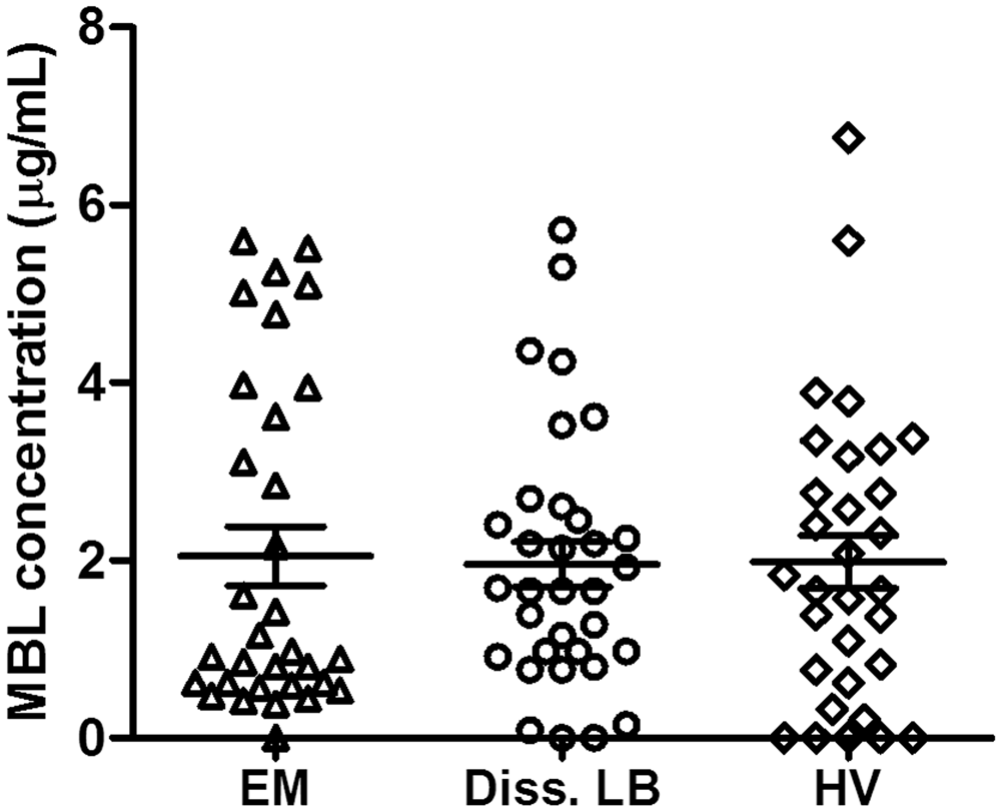
MBL levels in Lyme borreliosis patients versus healthy controls. MBL levels in serum from Lyme borreliosis (LB) patients with confirmed erythema migrans (EM) or disseminated LB (Lyme neuroborreliosis or acrodermatitis chronica atrophicans (LNB + ACA) and in healthy controls previously collected at our institute were measured using an ELISA coated with mannan. Bound serum MBL was detected by a mouse anti- human MBL antibody. The mean of MBL levels in serum from 31 patients with EM was 2.04 µg/mL and in 33 patients with LNB or ACA 1.96 µg/mL, compared 1.98 µg/mL in 31 healthy controls. Percentages of MBL deficiency per group (determined as <500 µg/mL) were 15.6% (n = 5) for erythema migrans, 12.1% (n = 4) for LNB and ACA, and 22.6% (n = 7) for healthy controls. Error bars represent the mean ± SEM.

Others have correlated MBL deficiency to be more prevalent in individuals with detectable *B. burgdorferi* s.l. antibodies in serum^19^. To further investigate whether MBL deficiency indeed plays a role in the risk of contracting *B. burgdorferi* s.l. infection or LB, we compared MBL levels in serum from LB patients to those of healthy controls. MBL levels were measured by an enzyme-linked immunosorbant assay (ELISA) of 31 patients with erythema migrans (localized disease), 33 patients with neuroborreliosis or ACA (late or disseminated disease), and 31 healthy controls. We found comparable levels of MBL in all three groups (Fig. 1).

### MBL deficient mice harbor higher dermal *B. burgdorferi* loads compared to WT mice

**Figure 2:**
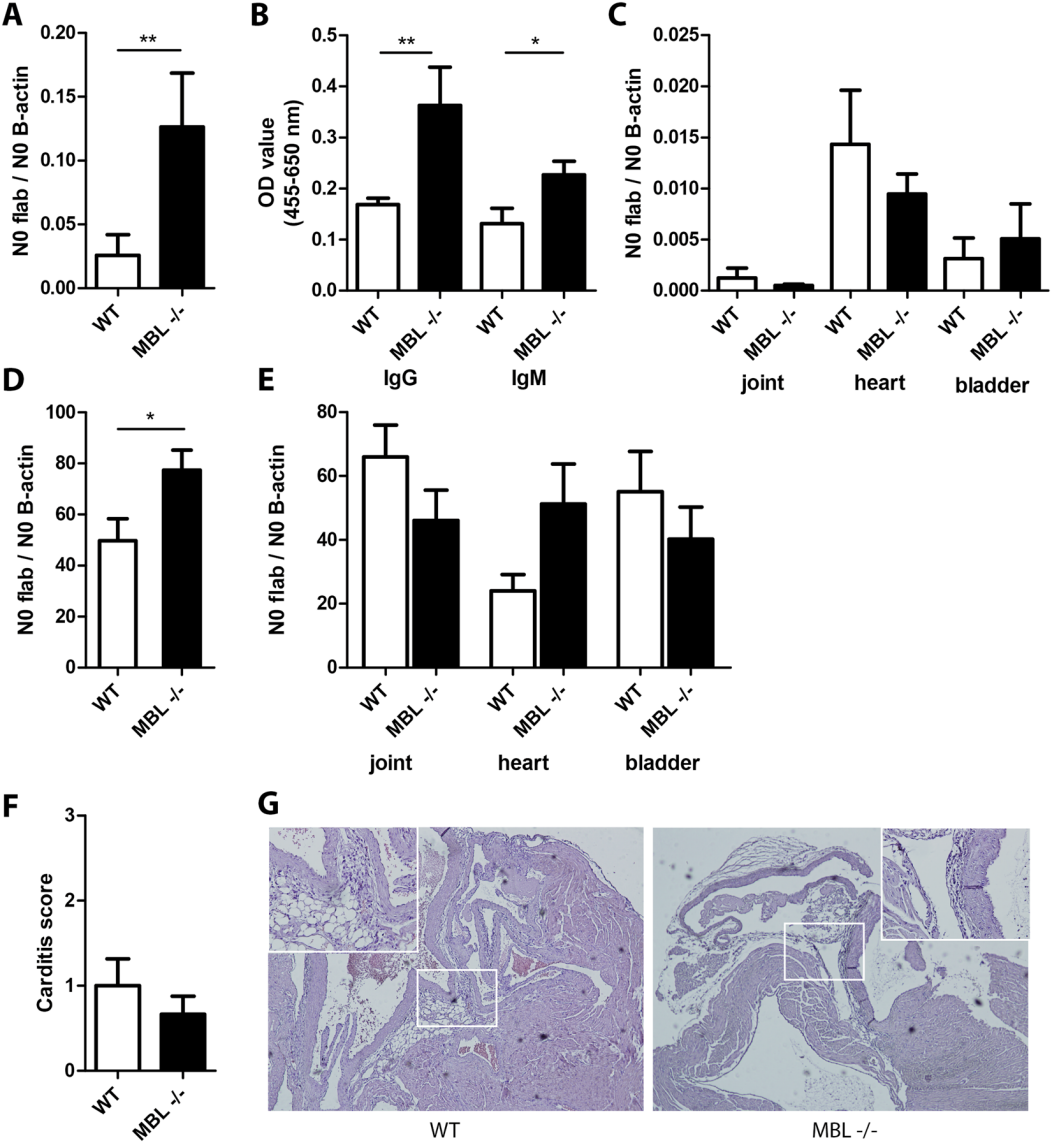
Increased *B. burgdorferi* burden in skin tissue but not in other tissues of MBL deficient mice during murine *B. burgdorferi* infection. (**A**–**C**) WT and MBL deficient mice (8 per group) were infected with 10^6^ *B. burgdorferi* N40 via subcutaneous inoculation. Mice were sacrificed after 14 days and assessed for *B. burgdorferi* burden. DNA was extracted using the Qiagen Blood and Tissue kit from skin, heart, joint and bladder and were subjected to quantitative *B. burgdorferi flaB* PCR normalized for quantitative mouse *β-actin* PCR. A. qPCR assessment of *B. burgdorferi* burden in skin after 14 days. (**B**) IgG and IgM titers against *B. burgdorferi* lysate in serum measured by ELISA. (**C**) qPCR assessment of *B. burgdorferi* burden in joint, bladder or heart. (**D,E**) WT and MBL deficient mice (8 per group) were infected with *B. burgdorferi* N40 via a tick challenge (4–5 ticks/mouse). Two separate experiments were performed, one with three animals per group and one with five animals per group. *B. burgdorferi* burden was normalized to the highest WT control value per experiment to pool both experiments. (**D**) qPCR assessment of *B. burgdorferi* burden in skin from ear biopsy at 7 days. (**E**) qPCR assessment of *B. burgdorferi* burden in skin, joint, bladder or heart at 21 days. (**F,G**) Five µm-thick sagittal heart sections were stained with hematoxylin and eosin. A carditis score was performed by an independent pathologist who was blinded to the experimental design on a scale from 0–3 with 0 being no, 1 mild, 2 moderate, and 3 being severe carditis. Carditis was characterized by disperse inflammation at the atrioventricular junction and aortic root. See Fig. S1 for a normal heart from a mice injected with PBS as a control. Error bars represent mean ± SEM and mean values significantly different in a two-tailed non-parametric Mann-Whitney test are indicated by asterisks (*p < 0.05 and **p < 0.01).

To more specifically determine the role of MBL during *B. burgdorferi* s.l. infection, we compared *B. burgdorferi* survival in the murine host using MBL deficient C57BL/6 mice and WT C57BL/6 mice. Mice were infected subcutaneously with 10^6^ *B. burgdorferi* N40 and after 14 days of infection *B. burgdorferi* infection in skin and disseminated organs was measured by quantitative Polymerase Chain Reaction (qPCR). In addition, we determined antibody responses against *B. burgdorferi* in serum. Interestingly, mice lacking MBL had significantly higher loads of *B. burgdorferi* in skin after 14 days (Fig. 2A). Furthermore, higher titers of anti-*B. burgdorferi* IgG and IgM were observed in serum of MBL deficient mice compared to WT mice (Fig. 2B). However, *B. burgdorferi* numbers in deeper tissue (heart, joint and bladder) were similar between both mouse strains (Fig. 2C). Culture of skin and bladder confirmed live spirochetes but showed no difference between WT and mice lacking MBL (Supplemental Table 1). To support these findings, we also challenged WT and MBL deficient mice with *B. burgdorferi* N40-infected *I. scapularis* nymphs in an independent experiment (Fig. 2D,E). There were no significant differences in post-feeding tick weights or *B. burgdorferi* burden in the ticks (data not shown). Interestingly, we again observed significantly higher loads of *B. burgdorferi* in MBL deficient mice at an early time point the tick bite site (Fig. 2D), but we did not find any differences in dissemination of *B. burgdorferi* to deeper tissue at a later time point between both mouse strains (Fig. 2E). Higher numbers of spirochetes, based on *flaB*/*B-actin* copies, were observed in mice infected by ticks compared to needle inoculation. We speculate this is because of a difference in viability and survival of *B. burgdorferi* spirochetes transmitted by ticks compared to *B. burgdorferi* spirochetes that have been grown *in vitro* in BSK medium. We also compared histological signs of inflammation in *B. burgdorferi-*infected organs in the mice infected by needle inoculation. Signs of arthritis were mild or absent and no differences between WT and MBL deficient mice were observed (data not shown). In contrast, all *B. burgdorferi*-infected mice developed carditis, but WT and MBL deficient mice had a similar score for inflammation (Fig. 2F,G). Control animals injected with PBS alone did not display carditis (Fig. S1).

### MBL binds to B. burgdorferi but is not crucial in killing of *B. burgdorferi* by murine serum

**Figure 3:**
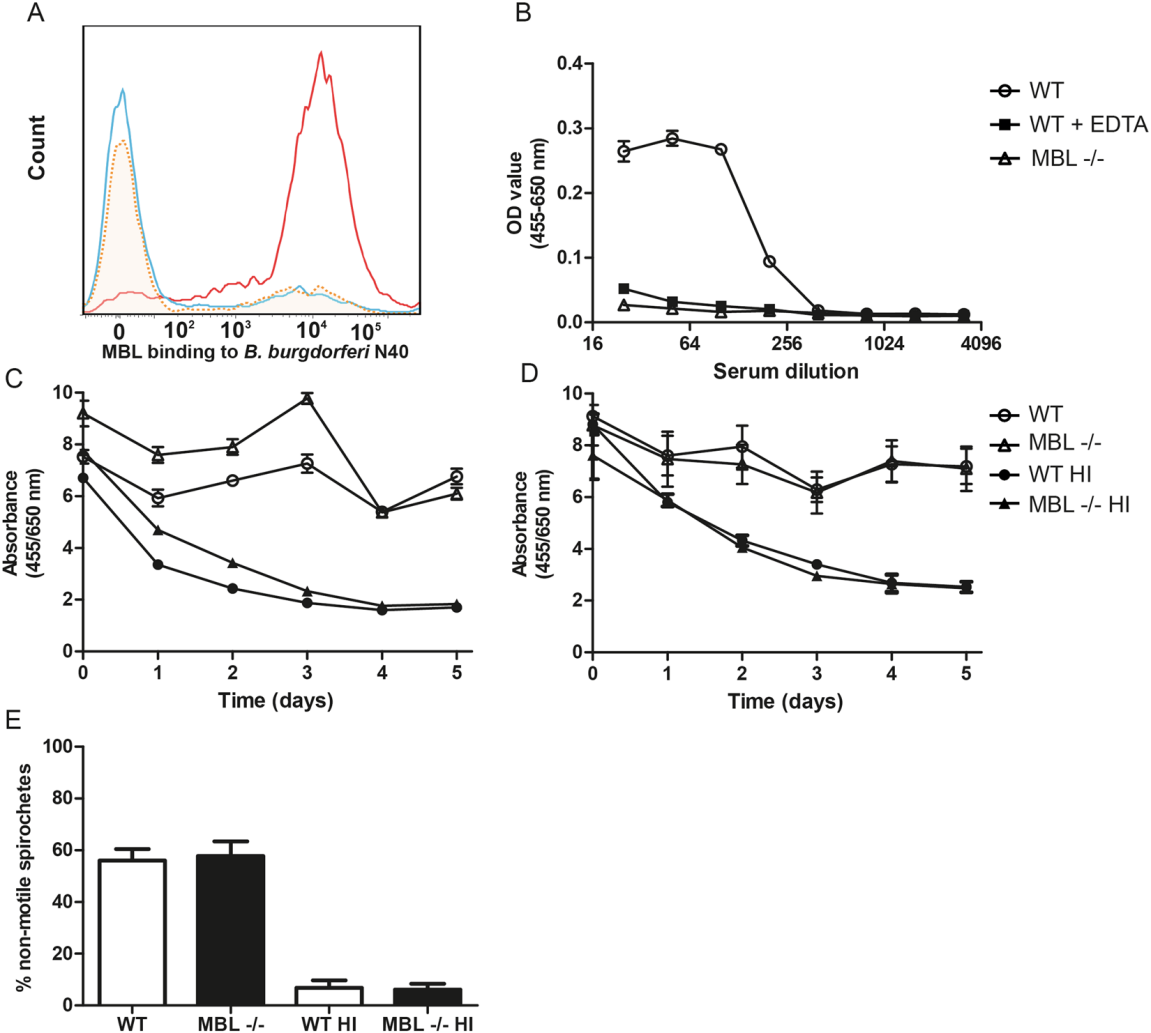
MBL binds to *B. burgdorferi*, but MBL deficiency in mice does not decrease complement-dependent killing of *B. burgdorferi*. (**A**) Recombinant human MBL (5 µg/mL) was added to cultured CFSE-labeled *B. burgdorferi* N40 spirochetes (2 × 10^8^ spirochetes/mL) and after two washing steps, binding of MBL to *B. burgdorferi* was subjected to FACS analysis (red line). As a control, 100 mM EDTA was added together with MBL which abrogates calcium dependent binding of MBL CRD’s (yellow dotted line). The blue line represents spirochetes without MBL. Binding of MBL was determined using a mouse anti-human MBL antibody and a goat anti-mouse IgG Texas Red antibody. (**B**) ELISA assessment of dose-dependent binding of MBL in murine serum to *B. burgdorferi* membrane protein extract-coated plates. WT serum or MBL deficient murine serum was added to wells and murine MBL was detected using an antibody against murine MBL-A. As a control, normal murine serum was pre-incubated with 100 mM EDTA. (**C**) Growth inhibition of *B. burgdorferi* strain N40 by murine serum was measured by the color change of Phenol Red at 562/630 nm once a day for up to five days. Ratio NMS/HI NMS and MBL deficient serum/HI MBL deficient serum after five days was 3.66 and 3.01, respectively. (**D**) Growth inhibition of *B. garinii* strain A87S by murine serum. The ratio NMS/HI NMS and MBL deficient serum/HI MBL deficient serum after five days was 2.84 and 2.86, respectively. (**E**) *B. garinii* strain A87S was incubated with 25% WT NMS, 25% MBL deficient murine serum or 25% heat-inactivated (HI) NMS (control). After 1 h of incubation the percentages of non-motile spirochetes were determined by a researcher blinded to the experimental design. Each sample and each time point were performed in triplicates and symbols or bars represent the mean ± SEM.

To elucidate how MBL deficiency results in higher *B. burgdorferi* survival, we performed several *in vitro* experiments to study the role of MBL in several innate immune responses against *B. burgdorferi*. We first determined whether MBL directly interacts with *B. burgdorferi*. Indeed, binding of recombinant human MBL to *B. burgdorferi* was observed using flow cytometry (Fig. 3A). This was abrogated in the presence of 100 mM EDTA, which suggests that MBL binds to *B. burgdorferi* in calcium dependent fashion via its carbohydrate recognition domains (CRDs). Next, we assessed whether murine native MBL was able to bind to *B. burgdorferi* membrane proteins using an ELISA coated with extracellular *B. burgdorferi* N40 extracts. We observed a dose-dependent increase in binding of MBL-A to extracellular *B. burgdorferi* extracts using normal murine serum (NMS), which we did not observe in MBL deficient murine serum or in NMS pre-incubated with 100 mM EDTA, suggesting that also MBL-A binds to *B. burgdorferi* membrane proteins via its CRDs (Fig. 3B).

We have previously shown that *B. burgdorferi* is partially protected from killing by human serum in the presence of a neutralizing antibody against human MBL, as well as in human MBL deficient serum^12^. We therefore hypothesized that murine serum lacking MBL would have impaired complement-dependent killing of *B. burgdorferi*. For this we used a growth inhibition assay, which has been used previously to study complement-dependent killing of *B. burgdorferi*^12,23^. *B. burgdorferi* N40 was incubated in 50% normal mouse serum (NMS) or 50% heat-inactivated (HI) mouse serum of either WT or MBL deficient mice. We found that active mouse complement was able to inhibit the growth of *B. burgdorferi* N40, as no decrease in absorbance was observed after five days in contrast to heat-inactivated serum (Fig. 3C). No difference in growth inhibition was however observed between MBL deficient and WT NMS (Fig. 3C). Next, we determined the percentage of dead (non-motile) spirochetes by dark-field microscopy after incubation of *B. burgdorferi* with serum at 37 °C. We demonstrated that all spirochetes remained motile after 1.5 h and 4 h of incubation with 50% WT NMS or MBL deficient serum using *B. burgdorferi* N40 (data not shown). Both experiments were also performed with *B. garinii* A87S, a strain which is known to be sensitive to complement-dependent killing. Growth inhibition by NMS was observed for *B. garinii* A87S, but this was again similar when MBL deficient serum was used (Fig. 3D). Dark-field microscopy showed that WT murine serum was indeed able to kill *B. garinii* spirochetes (Fig. 3E), however the percentage of non-motile *B. garinii* spirochetes by dark-field microscopy were comparable for both WT and MBL deficient serum. Together these data suggest that the higher *B. burgdorferi* loads observed in MBL deficient mice are unlikely to be due to impaired killing by the complement system in MBL deficient mice.

### MBL deficient mice have a similar inflammatory cytokine response upon stimulation with *B. burgdorferi* compared to WT mice

**Figure 4:**
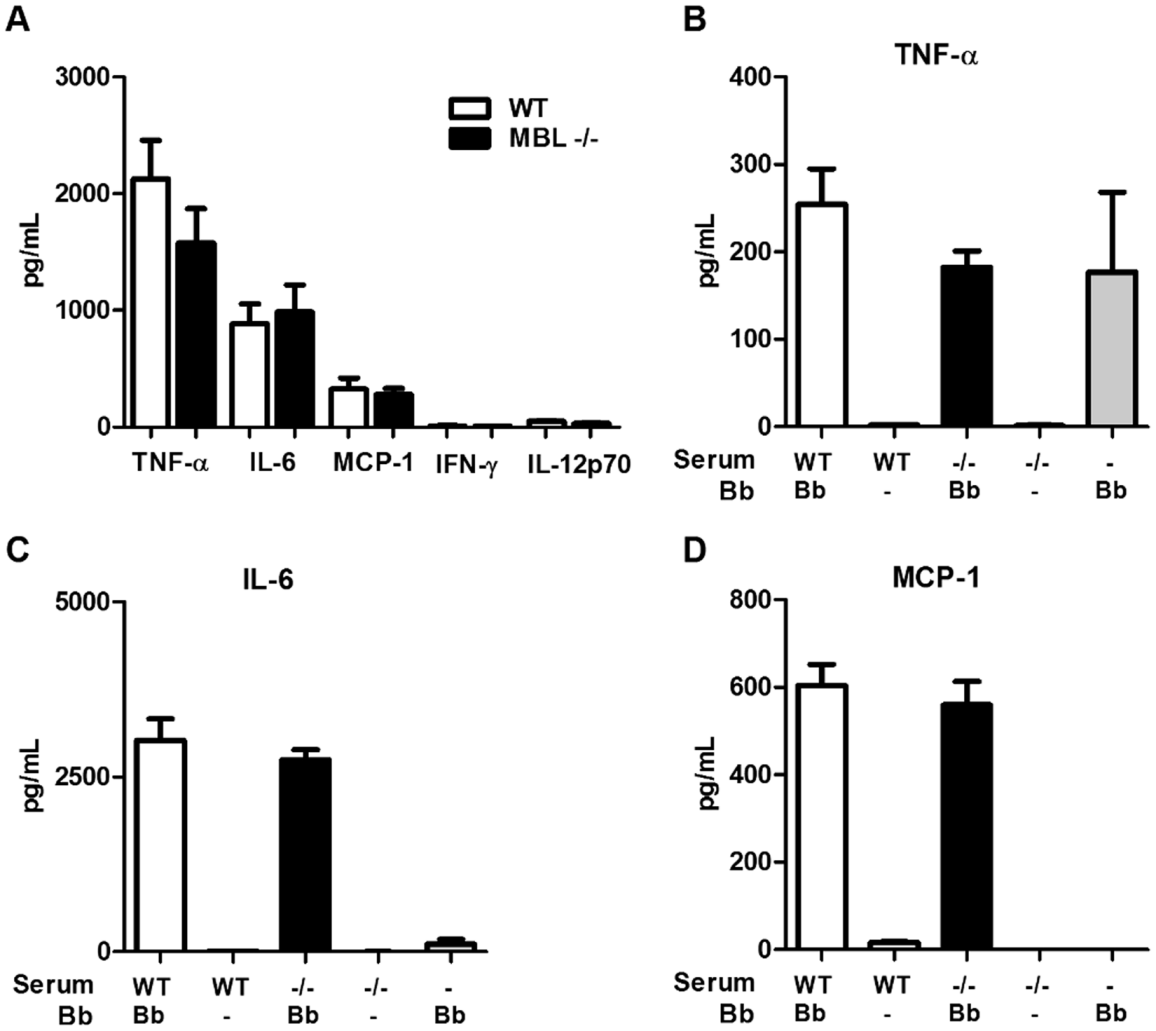
Levels of cytokines upon *in vitro* stimulation with *B. burgdorferi* N40 are similar between MBL deficient mice and WT control. (**A**) TNF-alpha, IL-6, MCP-1, IFN-y and IL-12p70 levels in the supernatant of murine whole blood stimulated with *B. burgdorferi* N40 (MOI: 5) for 16 h. Cytokines levels were measured using a mouse inflammation cytometric bead array (CBA), see also Table S2. As a control, whole blood was incubated in RPMI medium. Detection range: 2.5–10.000 pg/mL. (**B**,**D**) A similar experiment was performed with murine macrophages collected from peritoneal lavage fluid. 50.000 macrophages were stimulated with *B. burgdorferi* (MOI: 5) for 16 h in the presence of 20% WT serum or MBL deficient serum. Samples without serum and samples with serum only were used as control. Levels of TNF-alpha (**B**), IL-6 (**C**) and MCP-1 (**D**) are shown, IFN-y and IL-12p70 levels were below detection limit. Samples were performed in triplicates and the experiment was performed twice. Symbols or bars represent the mean ± SEM.

Since we did not find differences in complement-dependent killing between WT and MBL deficient murine serum *in vitro*, we hypothesized that lower pro-inflammatory and/or increased immunomodulatory cytokine responses could be responsible for the observed increased *B. burgdorferi* survival *in vivo*. Therefore, we assessed the levels of cytokines in the supernatant of murine whole blood upon stimulation with *B. burgdorferi* N40. Measurement of cytokine levels by cytometric bead array (CBA) after 16 h of incubation showed that levels of TNF-alpha, IL-6 and MCP-1 in whole blood were increased in the presence of *B. burgdorferi* compared to medium only, whereas IFN-gamma and IL-12p70 levels were below detection limit (Fig. 4A and Table S2 for the medium only). However, we did not find any differences comparing cytokine levels of MBL deficient mice to WT mice (Fig. 4A). These findings were corroborated using murine macrophages. In addition, WT peritoneal macrophages were collected and allowed to adhere overnight and incubated with *B. burgdorferi* in the presence of WT or MBL deficient serum. Comparing cytokine levels in the presence of 20% WT or MBL deficient serum to medium without serum showed that serum components were found to be crucial for the production of IL-6 and MCP-1, but not TNF-alpha, by murine macrophages upon stimulation with *B. burgdorferi* (Fig. 4B–D). IFN-gamma and IL-12p70 levels were again below detection limit (data not shown). However, in line with the whole blood experiments, we found similar levels of TNF-alpha, IL-6 and MCP-1 in the presence of WT or MBL deficient serum. Together these data indicate that the higher *B. burgdorferi* loads observed in MBL deficient mice are unlikely to be due to altered cytokine responses in MBL deficient mice.

### MBL deficiency does not play a role in *B. burgdorferi* phagocytosis by murine neutrophils or peritoneal macrophages

**Figure 5:**
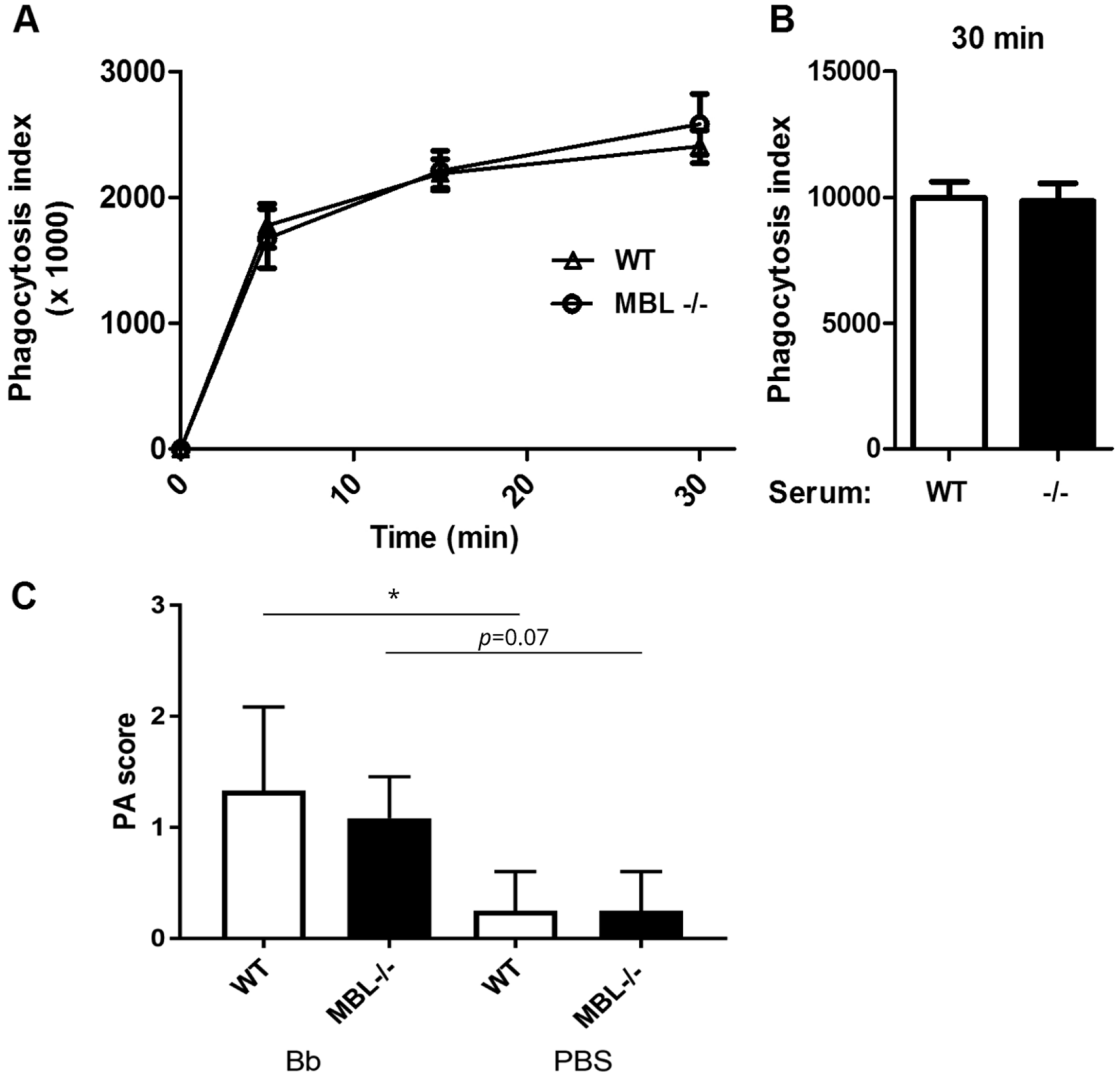
Phagocytosis of *B. burgdorferi* and inflammation *in vivo* during early infection are similar between MBL deficient mice and WT control. (**A**) Phagocytosis of *B. burgdorferi* by murine neutrophils in whole blood. Whole blood samples from five mice were subjected to FACS analysis after 5, 15 and 30 min of incubation at 37 °C with CFSE-labeled viable *B. burgdorferi* N40. Neutrophils were gated using rat-anti mouse Ly6 antibodies and the phagocytosis was calculated by the mean fluorescence x percentage of positive neutrophils (phagocytosis index). Samples kept on ice were used as baseline. (**B**) A second phagocytosis experiment was also performed with macrophages collected from peritoneal fluid from WT mice. Macrophages (50.000 per sample) were incubated with 10% WT serum or MBL deficient serum and *B. burgdorferi* N40 spirochetes (MOI:25). Phagocytosis of CFSE-labeled viable *B. burgdorferi* N40 by murine macrophages was determined by FACS analysis and expressed as phagocytosis index. Both the experiments in (**A,B**) are a representative of two independent experiments, samples were performed in triplicates. Bar or symbols represent the mean ± SEM (**C**). Inflammation based on scoring of H&E staining of skin 6 hours after inoculation of *B. burgdorferi*. WT: wildtype mouse, MBL −/−: MBL deficient mouse. Bb: experimental groups in which *B. burgdorferi* N40 was injected intradermally (10^6^ spirochetes per mice in 10 uL). PBS: control groups (n = 2) in which 10 uL PBS was injected intradermally. The experiment was performed twice. Error bars represent mean ± SEM.

Another function of the innate immune response by which MBL could be involved in the immune response against *B. burgdorferi* is phagocytosis by neutrophils or macrophages. Interestingly, we previously found that in the presence of TSLPI, phagocytosis of *B. burgdorferi* by human granulocytes was decreased^12^. To determine whether MBL deficiency would result in decreased phagocytosis, we collected murine whole blood from MBL and WT mice and added CFSE-labeled viable *B. burgdorferi* N40 to a multiplicity of infection (MOI) of 10. Phagocytosis of *B. burgdorferi* by murine neutrophils (Ly6 positive cells) after 5, 15 and 30 minutes at 37 °C was measured by flow cytometry, as previously described^12^. No significant difference in phagocytosis of *B. burgdorferi* N40 was observed between MBL deficient murine whole blood and WT murine whole blood (Fig. 5A). To support our findings, we also investigated opsonophagocytosis by WT murine macrophages collected from peritoneal lavage fluid in the presence of 10% serum from WT or MBL deficient mice. Although we observed a dose-dependent increase of phagocytosis in the presence of WT murine serum after 30 minutes of incubation at 37 °C (Fig. S2), we did not observe a difference in phagocytosis by WT murine peritoneal macrophages in the presence of WT or MBL deficient serum (Fig. 5B). Together these data indicate that the higher *B. burgdorferi* loads observed in MBL deficient mice are unlikely to be due to altered opsonophagocytosis in MBL deficient mice.

### Early inflammation *in vivo* during *B. burgdorferi* infection is not impaired by MBL deficiency

We next analyzed by an *in vivo* migration experiment whether the higher burden of *Borrelia* measured in murine skin in the absence of MBL is the result of an impaired inflammatory response in the early phase of a *Borrelia* infection. Therefore, we inoculated C57BL/6 WT and MBL deficient mice with *B. burgdorferi* or PBS as a control and harvested skin at 6 hours post infection. We scored inflammation in H&E staining of sagittal skin sections on a score of 0–3. In line with what we have previously described^24^, inoculation of spirochetes by itself resulted in inflammation, as can also be appreciated by comparison to the control groups. However, inoculating *Borrelia* resulted in a higher, but comparable, inflammation score for both WT and MBL deficient mice at t = 6 hours (Fig. 5C).

## Discussion

Previously we have shown that the lectin pathway, which can be activated by MBL, is important for the innate immune response against *B. burgdorferi*^12^. Inhibition of MBL by the tick protein TSLPI resulted in decreased complement-depended killing and phagocytosis by human neutrophils. Furthermore, others have found that MBL deficiency was more often present in individuals with antibodies against *B. burgdorferi* s.l. in serum compared to *B. burgdorferi* s.l. seronegative individuals^19^. However, the presence of antibodies does not equal disease. Therefore, we determined MBL levels in Lyme borreliosis patients and healthy controls and we showed that levels of MBL in patients with active *B. burgdorferi* s.l. infection were similar to MBL levels in healthy controls, although it should be mentioned we have only investigated a small number of patients and controls. In our opinion, it still remains to be determined whether MBL deficiency in humans is truly associated with a higher risk of infection with *B. burgdorferi* s.l. It could also be that impaired recognition of *B. burgdorferi* s.l. in MBL deficient individuals protects them against developing (severe) LB manifestations. This duality has been shown for TLR-2, where a heterozygous Arg753Gln polymorphism was found to result in a decreased *in vitro* immune response against *B. burgdorferi* N40 lysate and this polymorphism was less often present in patients with late stage LB^25^.

To further elucidate the role of MBL during *B. burgdorferi* infection *in vivo*, we performed experiments with WT mice and mice that lack MBL. We demonstrated that murine MBL is able to bind to *B. burgdorferi*, and that compared to WT mice MBL deficient mice have higher *B. burgdorferi* loads in skin tissue at the inoculation or tick bite site. Besides higher dermal loads we also found higher anti-*B. burgdorferi* antibody titers in serum of MBL deficient mice, which are known to correlate well with spirochetal burden^26^. However, no increase in *B. burgdorferi* loads in MBL deficient mice were observed in deeper tissue such as joint, heart and bladder. We have used *B. burgdorferi* N40 for both the needle inoculation and tick infestations experiments, which is a strain capable of inhibiting complement activation. It could be that MBL deficiency has a more significant impact on the survival of serum sensitive *B. burgdorferi* s.l. species such as *B. garinii*, but serum-sensitive *B. garinii* strains are, to the best of our knowledge, not able to establish an infection in immunocompetent laboratory mice. Indeed, in our hands *B. garinii* strain ZQ1, previously shown to be infectious in severe combined immunodeficiency (SCID) mice^27^, even at an inoculum as high as 10^7^ spirochetes, was not capable of causing an infection in MBL deficient or WT C57BL/6 mice (data not shown). Identification of a serum sensitive strain capable of murine infection would be of great value for research on the role of complement during *Borrelia burgdorferi* s.l. infection.

MBL deficiency has also been linked to decreased disease burden as the result of decrease immune responses^18^. However, comparing inflammation in WT and MBL deficient mice, we found no differences in inflammation of skin six hours after inoculation. This suggests that influx of immune cells to the site of *B. burgdorferi* infection is not impaired in MBL deficient mice and can thus not explain the higher *B. burgdorferi* loads in skin at early time points in these mice compared to WT controls. In addition, inflammation in heart tissue was similar in both WT and MBL deficient animals 14 days after *B. burgdorferi* syringe inoculation. We chose to work with the C57BL/6 genetic background based on the availability for MBL knockout mice. Importantly, the C57BL/6 genetic background of the mice is a genetic background known to be less susceptible for inflammation caused by *B. burgdorferi* infection^28^. Indeed, we could hardly find any signs of inflammation in skin or joint tissue (data not shown), despite confirmation of *B. burgdorferi* by qPCR and culture in these tissues. In the future, backcrossing MBL deficient mice to a more *B. burgdorferi-*susceptible C3H/HeN background could reveal whether MBL deficiency would result in a reduced inflammatory response during *B. burgdorferi* infection, although the extent of carditis was similar in WT and MBL deficient mice.

We have performed multiple *in vitro* experiments to identify the mechanism behind the observed increased *B. burgdorferi* survival at early time points in skin from mice lacking MBL. *In vitro* experiments using murine serum did not show impaired killing of a complement-sensitive *B. garinii* isolate by MBL deficient serum, as compared to WT serum. In addition, in our hands both WT and MBL deficient serum was able to inhibit outgrowth of the more complement-resistant *B. burgdorferi* N40, yet neither serum caused killing of *B. burgdorferi* N40 spirochetes as determined by dark field microscopy. Furthermore, cytokine production upon stimulation with *B. burgdorferi*, and phagocytosis by murine neutrophils in whole blood or by peritoneal macrophages was similar for WT and MBL deficient mice.

The discrepancy between human serum and murine serum in our previous and current experiments might be explained by the instability of murine complement, as described in literature^29^. Although we have taken the utmost precautions while obtaining and working with murine serum, i.e. freeze-thawing was avoided and serum was processed within a few hours after collection - the instability of murine complement, might have complicated our experiments. Murine complement instability might (partially) explain why murine MBL deficiency is beneficial for *B. burgdorferi* survival *in vivo*, but not *in vitro*. Another explanation for the observed limited role of MBL is the fact that the complement system is an intricate system consisting of many different pathways that can initiate other innate immune responses. It could be that role of the lectin complement pathway is less important than the alternative or classical pathway in complement-mediated killing of *Borrelia* spirochetes, or that these pathways take over the role of the lectin pathway by initiating other innate immune responses in MBL deficient mice. Also, even within the lectin pathway itself, other components than MBL, such as ficolins or MASP-2, could have a compensatory role in the activation of the lectin pathway. This is supported by observations that TSLPI was also able to protect *B. burgdorferi* by inhibiting not only human MBL, but also L-ficolin. Further research is necessary to elucidate the role of MBL during *B. burgdorferi* infection as well as mechanisms that compensate innate immune responses in the absence of MBL.

To conclude, we assessed the role of MBL during *B. burgdorferi* infection *in vivo* and showed that MBL plays a role in survival of *B. burgdorferi* in the skin of the murine host *in vivo*. Our *in vitro* observations indicate that MBL is not crucial for murine complement dependent killing, cytokine production or phagocytosis of *B. burgdorferi* s.l. and we did not find evidence that influx of immune cells is impaired in MBL deficient mice compared to WT mice. Thus, the mechanism by which MBL facilitates early clearance of *B. burgdorferi* warrants further investigation. Further research should focus on other complement factors that activate or modulate the lectin pathway.

## Material and Methods

### Mice

Experiments with ticks and mice were performed at Yale University, New Haven USA. All other animal experiments were performed at the Academic Medical Center (AMC) at Amsterdam, the Netherlands. Specific pathogen-free wildtype C57BL/6 mice and MBL-A and -C deficient C57BL/6 mice were purchased from Jackson Laboratories (Bar Harbor, ME)^20^. At the AMC, MBL null mice were bred in the animal facility of the Academic Medical Center (Amsterdam, The Netherlands). Age-and sex-matched animals were used in each experiment and the Animal Care and Use Committee of the University of Amsterdam approved all experiments (protocol number DIX102835). At Yale University, the animal experimental protocol was approved by the Yale University’s Institutional Animal Care & Use Committee (protocol number 2008–07941, approval date: 3/31/2014). All experiments were performed in accordance with relevant guidelines and regulations.

### MBL serum levels in LB patients and healthy controls

Serum from LB patients with confirmed LB manifestations (EM, neuroborreliosis or ACA) or healthy controls previously collected at our institute were utilized for this analysis^3,30^. Wells (Microlon, Greiner) were coated with 10 mg/mL mannan overnight at 4 °C, blocked with 1% BSA in PBS. After blocking wells, serum dilutions (1:50 and 1:100) in wash buffer (TBS/Tween 20/CaCl_2_) were added and incubated overnight at 4 °C. Wells were washed and incubated with monoclonal mouse anti-human MBL antibody (10 µg/mL, Sanquin, the Netherlands) in wash buffer for 1 hr at RT. After washing, HRP-conjugated anti-mouse IgG (1:4000) was added and incubated for 45 min at RT. The amount of MBL bound to the plates was determined by reading the absorbance at 450/655 nm using the iMark Microplate Reader (Bio-Rad). Concentrations were calculated by interpolation from a standard curve. 500 ng/mL was used as a cut-off for MBL deficiency^15^. All samples were tested in duplicate.

### Murine *B. burgdorferi* infection by needle inoculation

To study the role of MBL *in vivo*, *B. burgdorferi* N40 spirochetes that were previously recovered from a bladder from a experimentally infected mice, were cultured in modified Kelly medium (MKM) and 10^6^ spirochetes in PBS were inoculated in the midline of the back, as described previously^24^. As a control, four mice were inoculated with only PBS. Mice were sacrificed at two weeks post-infection and skin (inoculation site), bladder, heart and tibiotarsi were collected and saved for histopathological examination, culture or qPCR, as described previously^24^. Serum was collected (see below) for IgG and IgM assessment. DNA from the organs was extracted using the Qiagen Blood and Tissue kit and *B. burgdorferi* loads were subjected to quantitative *B. burgdorferi flaB* PCR normalized for quantitative mouse β-actin PCR. To assess carditis, five µm-thick paraffin embedded sections of sagittally dissected hearts were processed and stained with hematoxylin and eosin by routine histological techniques. Carditis was scored on a scale from 0 to 3 by a pathologist blinded to the experimental design, as previously described^24^.

### Detection of *B. burgdorferi* IgG and IgM levels in murine serum

*B. burgdorferi* N40 specific IgG and IgM in murine serum from mice infected with *B. burgdorferi* N40 were measured by ELISA, as described previously^24^. Briefly, wells were coated with *B. burgdorferi* N40 lysate (1 µg/mL) and bound IgG and IgM was detected with respectively anti-mouse IgG and anti-mouse IgM HRP (both 1:2000). Serum from mice injected with PBS was used as a control and all measurements were performed in duplicate.

### *B. burgdorferi* infection by I. scapularis tick infestation

Four *B. burgdorferi* N40 infected nymphs per mice were placed on eight MBL deficient or eight WT control mice, as described previously^31^. Nymphs were allowed to feed to repletion. DNA was isolated from skin punch-biopsies at day 7 and 21 and from heart and joints at day 21. *B. burgdorferi* burden was assessed by qPCR, as described above. Two separate experiments were performed, one with three animals per group and one with five animals per group and the results were pooled.

### Binding of recombinant human MBL to *B. burgdorferi*

Binding of MBL to *B. burgdorferi* N40 was essentially performed as previously described^32^. Cultured 2 × 10^8^ spirochetes/mL *B. burgdorferi* N40 were incubated with 5 µg/mL recombinant human MBL (R&D systems, Minneapolis, MN) for 30 minutes at 37 °C in HBSS with 5 mM CaCl_2_ and 5 mM MgCl_2_. As a control, medium with 100 mM EDTA was used. Spirochetes were washed and bound MBL was detected using mouse anti-human MBL (1:250) (mAb 3E7, Hycult-biotech, Uden, the Netherlands) and goat anti-mouse IgG Texas Red (1:1000, Invitrogen, CA, USA). Spirochetes were fixated using 4% PFA and MBL binding was measured by flow cytometry performed on a FACS Canto (Becton Dickinson).

### Binding of murine MBL to *B. burgdorferi* lysate

*B. burgdorferi* membrane extract^33^ was coated (10 µg/mL) on high binding microtiter plates (Microlon, Germany) overnight. Wells were blocked with PBS/1% BSA at RT for 1 h and incubated with normal murine serum, MBL deficient murine serum or normal murine serum pre-incubated with 100 mM EDTA, diluted from 1:25 to 1:3200 in PBS/0.05% Tween20/1% BSA. After 1 h, cells were washed and incubated with 1:500 rat anti-MBL-A IgG (mAb 8G6, Hycult-biotech, Uden, the Netherlands) and bound antibody was detected using HRP-conjugated anti-rat IgG (H&L) (Abcam, United Kingdom) and TMB as substrate (Thermoscientific, IL).

### Killing of *B. burgdorferi* and B. garinii by murine serum

Murine serum was collected from blood of WT and MBL deficient C57BL/6 mice via cardiac puncture. Since murine serum is highly unstable, experiments were started within 2 h after blood was collected in glass tubes at RT. Thirty minutes after collection, blood was centrifuged at 3000 g for 7 minutes and serum was collected and centrifuged again at 20000 g for 5 minutes at RT as previously described^29^. Complement-dependent growth inhibition was assessed, as previously described^12,23^. Briefly, *B. burgdorferi* N40 or *B. garinii* A87S (2 × 10^7^) were resuspended in 100 μl BSK-H medium containing 240 μg/mL phenol red with 50% murine serum (WT or MBL deficient serum) for 2 h at 37 °C. As a control, *B. burgdorferi* was resuspended in with 50% heat-inactivated murine serum. Samples were subsequently incubated at 33 °C for five days and growth of *B. burgdorferi* was determined by measuring the OD at 562/630 nm each 24 h using the Biotek Synergy HT plate reader. Each sample was performed in triplicates and the experiment was performed twice. For assessment of dead (non-motile) spirochetes by dark-field microscopy, spirochetes were added to a 96 well cell culture plate (10^7^/mL) and supplemented with 25% serum (*B. garinii* A87S) or 50% serum (*B. burgdorferi* N40), incubated at 37 °C and a 5 μL sample was observed under dark field microscopy, by a researcher blinded for the experimental design, for spirochete motility as described earlier^34^. Heat-inactivated serum was used as a control. Each sample was performed in triplicates and the experiment was performed three times.

### *In vitro* inflammatory cytokine response upon stimulation with *B. burgdorferi*

Whole blood from five naive MBL knock-out or WT mice were obtained from cardiac puncture and was collected in heparinized tubes. Whole blood was stimulated in 96-well microtiter plates (Greiner) with *B. burgdorferi* N40 (MOI: 5) suspended in RPMI medium with FCS and L-glutamine. RPMI medium without spirochetes was used as a control. Samples were incubated for 16 h at 37 °C and 5% CO_2_. Supernatants were collected and cytokine production was measured by a mouse inflammation cytometric bead array (BD, NJ, USA). For the cytokine production by murine macrophages, peritoneal macrophages were harvested from WT mice by peritoneal lavage as described elsewhere^35^. Adherent peritoneal macrophages (50.000) in Greiner 96-well plates were washed with RPMI twice. *B. burgdorferi* was added to a MOI of 5 in RPMI with 20% WT serum or MBL deficient serum (pooled sera from five mice). Samples without serum and samples with serum but without spirochetes were used as an control. Samples were incubated for 16 h at 37 °C and 5% CO_2_. Supernatants were collected and cytokine production was measured by CBA. All samples were performed in triplicates and both experiments were performed twice.

### Phagocytosis by whole blood or murine macrophages

Phagocytosis assays were performed in essence as described before^12,24^. Murine whole blood obtained from cardiac puncture from was collected in heparinized tubes. CFSE-labeled viable *B. burgdorferi* (3 × 10^8^ spirochetes/mL in 25 µL, MOI:10) was added to 125 µL murine whole blood. All materials were kept on ice until all samples were ready. Samples were incubated in a 37 °C water bath and at 5, 15 and 30 minutes 30 µL from each sample was added to 4 mL ice-cold FACS buffer (PBS, 5% bovine serum albumin (BSA), 0,01% NaN_3_ and 0,35 mM EDTA). Samples continuously kept on ice were used as a control. Subsequently, samples were incubated in erythrocyte lysis buffer for 20 minutes. Samples were washed and incubated with anti-Ly6-PE antibodies for 30 minutes. Phagocytosis of *B. burgdorferi* by neutrophils were visualized on the FACS Canto (Beckton Dickinson) and the phagocytosis index of neutrophils in each sample was calculated as mean fluorescence intensity (MFI) × percentage CFSE-positive at 37 °C minus the phagocytosis index of samples kept on ice. Each sample and each time point were performed in triplicates and the experiment was performed twice. Phagocytosis by peritoneal macrophages (see above) was assessed in essence as described above. Adherent peritoneal macrophages (50.000) in Greiner 96-well plates were washed with RPMI and stimulated with CFSE-labeled *B. burgdorferi* N40 (MOI: 25) in the presence of 10% WT serum or MBL deficient serum. After 30 minutes, the supernatant was removed and cells were washed twice, collected and processed for analysis by flow cytometry. Samples kept on ice were used as a control. The experiment was performed in triplicate.

### *In vivo* migration in murine skin

To assess influx of immune cells at the site of infection, we intradermally injected C57BL/6 WT and KO mice with 1 × 10^6^ *B. burgdorferi* in PBS in the midline of the neck and mice were sacrificed 6 hours post inoculation, as previously described^24^. Control animals were injected with PBS. The experiment was performed twice, one time with 8 mice per group and 4 controls and one time with 6 mice per group and 2 controls. Skin was harvested, formalin fixed and imbedded in paraffin. Five µm-thick sagittal skin sections were processed and H&E stained by routine histological techniques. Slides were scored on a scale from 0–3 for inflammation by an independent pathologist who was blinded to the experimental, with 0 being no, 1 mild, 2 moderate, and 3 being severe diffuse infiltration. Of each mouse, the average score of two slides was used.

### Statistical analysis

The significance of the difference between the mean values of the groups was analyzed using Prism 5.0 software (GraphPad Software, San Diego, CA). A two-tailed non-parametric Mann-Whitney test was used and a p-value of < 0.05 was considered significant.

## Supplementary information

Supplementary info

## Data Availability

The datasets generated during and/or analysed during the current study are available from the corresponding author on reasonable request.
