# Supplementary material for: The role of Mannose Binding Lectin in the immune response against Borrelia burgdorferi sensu lato
Source: Sci Rep. 2019 Feb 5;9:1431. doi: 10.1038/s41598-018-37922-8 (PMC6363739; doi:10.1038/s41598-018-37922-8)
Supplement: Supplementary file 1 — Supplementary info [file 41598_2018_37922_MOESM1_ESM.docx]

**The role of Mannose Binding Lectin in the immune response against *Borrelia burgdorferi* sensu lato**

Jeroen Coumou^1*^, Alex Wagemakers^1^, Sukanya Narasimhan^2^, Tim J. Schuijt^1^, Jasmin. I. Ersoz^1^, Anneke Oei^3^, Onno J. de Boer^4^, Joris J. T. H. Roelofs^4^, Erol Fikrig^2^ and Joppe W. Hovius^1^

^1^ Amsterdam UMC, University of Amsterdam, Center for Experimental and Molecular Medicine, Meibergdreef 9, Amsterdam, Netherlands.

^2^ Department of Internal Medicine, Yale University School of Medicine, 06511 New Haven, CT, USA.

^3^ Amsterdam UMC, University of Amsterdam, Department of Medical Microbiology, Meibergdreef 9, Amsterdam, Netherlands.

^4^ Amsterdam UMC, University of Amsterdam, Department of Pathology, Amsterdam Cardiovascular Sciences, Amsterdam Infection & Immunity, Meibergdreef 9, Amsterdam, Netherlands.

## Supplemental figures and tables


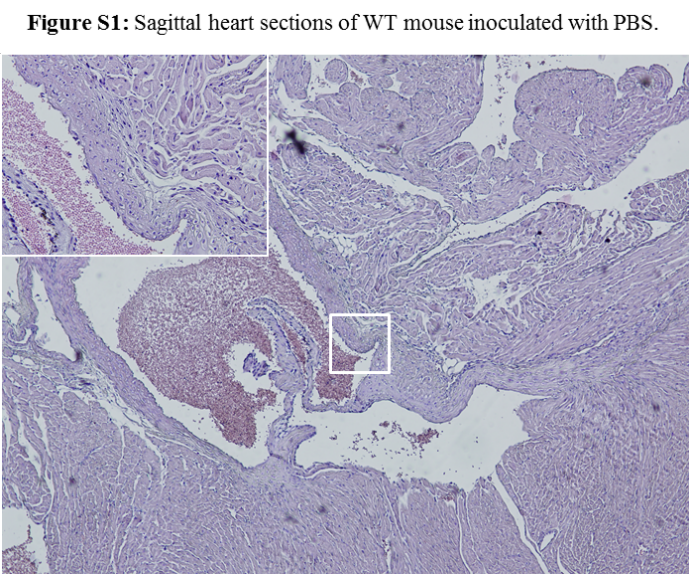


**Figure S1. Five µm-thick sagittal heart sections were stained with hematoxylin and eosin.** A carditis score was performed by an independent pathologist who was blinded to the experimental design on a scale from 0-3 with 0 being no, 1 mild, 2 moderate, and 3 being severe carditis. Carditis was characterized by disperse inflammation at the atrioventricular junction and aortic root.


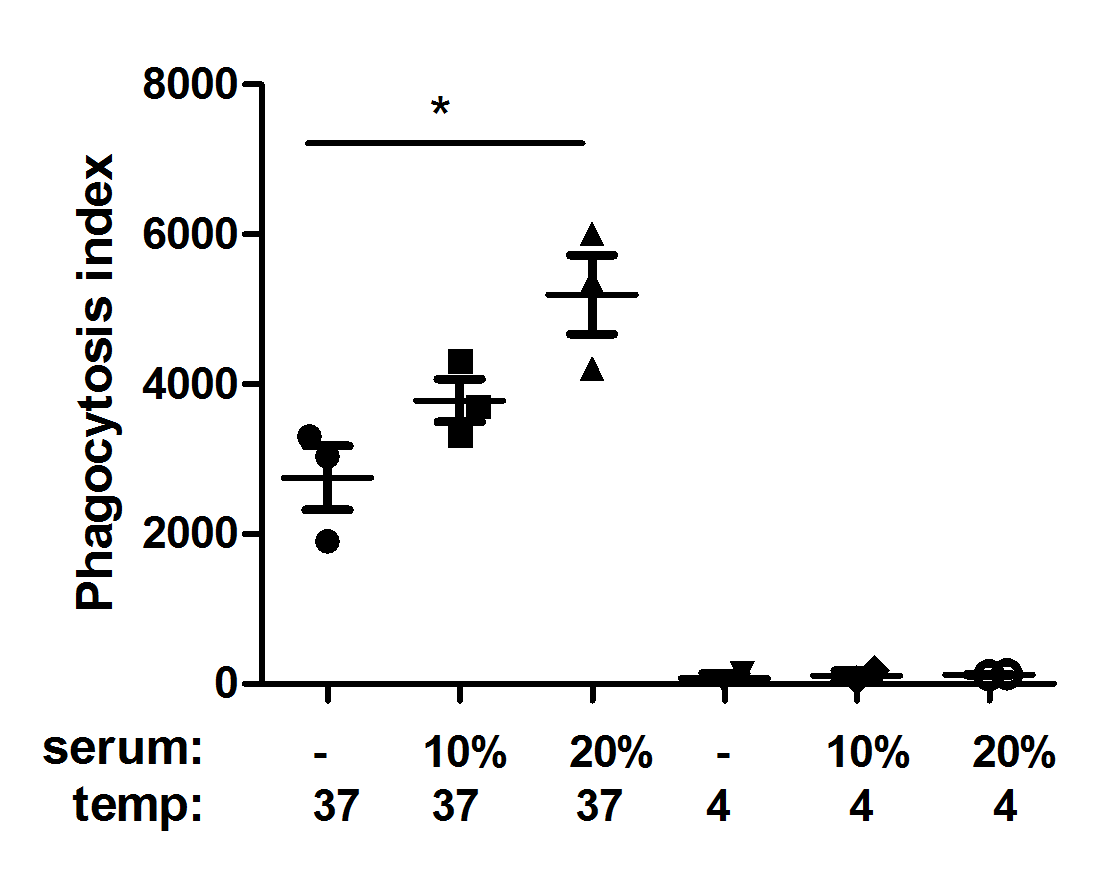


**Figure S2. Pilot experiment to determine optimal serum concentration for phagocytosis of *B. burgdorferi* by by murine macrophages.** Macrophages were collected from peritoneal fluid from WT mice. Macrophages (50.000 per sample) were incubated with WT serum or MBL deficient serum and *B. burgdorferi* N40 spirochetes (MOI:25). Phagocytosis of CFSE-labeled viable *B. burgdorferi* N40 by murine macrophages was determined by FACS analysis and expressed as the phagocytosis index. Error bars represent mean ± SEM and mean values significantly different in a Student’s t-test are indicated by asterisks (* p < 0.05).

**Supplemental Table 1. Culture of *B. burgdorferi* from murine tissue.**

| **weeks after sacrifice** | **positive cultures in skin** | **positive cultures in bladder** |
| --- | --- | --- |
| 1 | WT: 8/8  MBL -/-: 8/8 | WT: 0/8  MBL -/-: 0/8 |
| 3 | X  X | WT: 7/8*  MBL -/-: 5/8* |

WT and MBL deficient mice (8 per group) were infected with 10^6^ B. burgdorferi N40 via subcutaneous inoculation. Mice were sacrificed after 14 days and assessed for B. burgdorferi burden by culture. In intervals of 1 week, cultures were examined for live spirochetes under dark field microscopy. * In the WT group, 1 bladder sample got contaminated, in the MBL deficient group 2 bladder samples got contaminated and in tissue from 1 mice, no spirochetes were seen.

| **Supplemental Table 2. Cytokine levels measured in supernatant of murine whole blood from WT or MBL deficient mice** | | | | | | |
| --- | --- | --- | --- | --- | --- | --- |
|  | **Medium only** | | | ***B. burgdorferi* N40** | | |
|  | **WT** | **MBL -/-** | ***p-value*** | **WT** | **MBL -/-** | ***p-value*** |
| **TNF-α** | 6 (2-10) | 2 (1-4) | 0.08 | 11055 (8869-13242) | 13464 (9353-16044) | 0.82 |
| **IL-6** | 3 (<-3) | 6 (<-21) | 0.39 | 662 (67-1008) | 327 (190-571) | 0.19 |
| **IL-10** | < | < |  | 201 (68-371) | 121 (9-371) | 0.41 |
| **MCP-1** | < | < |  | 23 (< - 80) | 28 (<-128) | 0.87 |
| **IFN-y** | < | < |  | < | < |  |
| **IL-12p70** | < | < |  | < | < |  |
| Mean values (range in parenthesis) are shown. All values are in pg/mL.  < : below detection limit (2.5 pg / mL). | | | | | | |
